# The Influence of a Terminal Chlorine Substituent on the Kinetics and the Mechanism of the Solvolyses of *n*-Alkyl Chloroformates in Hydroxylic Solvents

**DOI:** 10.3390/ijms21124387

**Published:** 2020-06-19

**Authors:** Malcolm J. D’Souza, Jeremy Wirick, Osama Mahmoud, Dennis N. Kevill, Jin Burm Kyong

**Affiliations:** 1 Department of Chemistry, Wesley College, Dover, DE 19901-3875, USA; jjwirick@umes.edu (J.W.); osama.mahmoud@email.wesley.edu (O.M.); 2 Department of Chemistry and Biochemistry, Northern Illinois University, DeKalb, IL 60115-2862, USA; 3 Department of Chemical and Molecular Engineering, Hanyang University, Ansan-Si, Gyeonggi-do, 15588, Korea; jbkyong@hanyang.ac.kr

**Keywords:** solvolysis, propyl chloroformate, 3-chloropropyl chloroformate, butyl chloroformate, 4-chlorobutyl chloroformate, Grunwald–Winstein equation, addition-elimination, ionization

## Abstract

A previous study of the effect of a 2-chloro substituent on the rates and the mechanisms of the solvolysis of ethyl chloroformate is extended to the effect of a 3-chloro substituent on the previously studied solvolysis of propyl chloroformate and to the effect of a 4-chloro substituent on the here reported rates of solvolysis of butyl chloroformate. In each comparison, the influence of the chloro substituent is shown to be nicely consistent with the proposal, largely based on the application of the extended Grunwald–Winstein equation, of an addition-elimination mechanism for solvolysis in the solvents of only modest solvent ionizing power, which changes over to an ionization mechanism for solvents of relatively high ionizing power and low nucleophilicity, such as aqueous fluoroalcohols with an appreciable fluoroalcohol content.

## 1. Introduction

A recent report [1] concerning the effect of a terminal chlorine substituent on the solvolysis of ethyl chloroformate (**1**) [2] to give a study of 2-chloroethyl chloroformate (**2**) allowed a parallel reaction rate study in a range of pure and mixed solvents, mainly with water as one component but also for 2,2,2-trifluoroethanol-ethanol (T-E) mixtures. The reaction rate study [3] with propyl chloroformate (**3**) in the same and similar solvents has also been reported, and new studies involving the solvolysis of 3-chloropropyl chloroformate (**4**), butyl chloroformate (**5**), and 4-chlorobutyl chloroformate (**6**) are reported in Table 1 of this manuscript. These studies can be combined with earlier studies of ethyl chloroformate (**1**) and 1-chloroethyl chloroformate (**7**) to assess the effect of a terminal chlorine introduced into the alkyl group upon the solvolysis rates of ROCOCl substrates, where R is ethyl, propyl, or butyl. The specific rates for a variety of solvolysis of **4**, **5,** and **6** are presented in Table 1, together with measures of solvent nucleophilicity (*N*_T_) and solvent ionizing power (*Y*_Cl_). The nature of and the use of *N*_T_ and *Y*_Cl_ scales are discussed later in the manuscript, and they are used in conjunction with specific rates within a linear free energy relationship (LFER) approach in a study of the effect of solvent variation on the reaction mechanism [4].

**Scheme 1:**
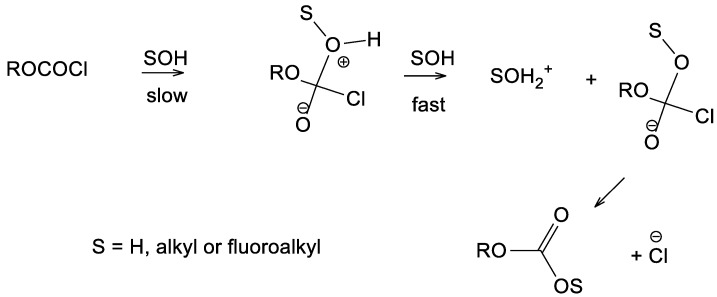
Addition-elimination (bimolecular mechanism).

**Scheme 2:**
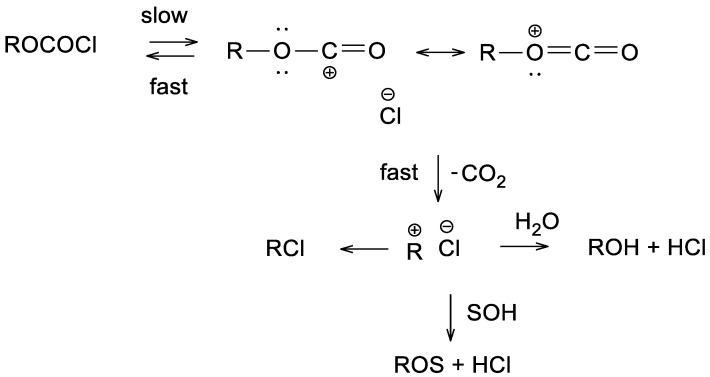
Ionization-elimination-addition (unimolecular mechanism).

Although minor adjustments could be made, the basic reaction mechanisms believed to operate in the solvolysis of chloroformate esters in the presence of a solvent, which can be represented as SOH, are shown in Scheme 1 and Scheme 2.

One aspect of the mechanisms summarized in Scheme 1 and Scheme 2 is that the introduction of electron supplying substituents into the R group favors the ionization pathway (Scheme 2), and electron-withdrawing substituents favor the rate-determining addition pathway (Scheme 1). As regards the SOH solvent, a more polar solvent, especially one capable of strong hydrogen bonding with the leaving chloride ion, favors the ionization pathway of Scheme 2, and a solvent with high nucleophilicity favors the rate-determining addition present in the Scheme 1 pathway.

In terms of the above discussion, we can see why it is found that aryl chloroformates, which would be disfavored by any mechanism requiring the formation of a very high energy phenyl cation (phenonium ion) [5,6,7,8,9,10,11], and methyl chloroformate, requiring the formation of a high energy methyl cation [12,13,14], react in all studied solvolytic reactions by the bimolecular mechanism (Scheme 1). In Scheme 2, the first formed intermediate, the resonance-stabilized carboxylium ion strongly favors a fast return to reactant rather than loss of CO_2_ to give the high-energy aryl or methyl cation, even in fluoroalchohol-water mixtures, which are very favorable towards ionization. The possibility of a concerted S_N_2 process for the bimolecular reaction has been given consideration, but Queen [6] presented strong evidence for an addition-elimination pathway.

An analysis of the rate data (Table 1) in terms of the extended Grunwald–Winstein equation follows. The simple equation was developed for application to reactions proceeding with a rate-determining ionization step, leading subsequently to substitution and/or elimination reaction [15], as expressed in Equation (1).

log (*k/k_o_*) = *mY* + *c* (1)

In Equation (1), *k* and *k*_o_ are the rates of reaction of a substrate in a given solvent and in the standard solvent, respectively. The standard solvent was arbitrarily chosen as 80% ethanol (80% ethanol-20% water, by volume at 25 °C), *m* is the sensitivity to changes in solvent ionizing power (the *Y*_Cl_ scale), which was initially based on the solvolysis of *t*-butyl chloride at 25 °C (*m* parameter arbitrarily set at unity for this solvolysis), and *c* is a constant residual term, usually of small value, unless the solvolysis proceeding in 80% ethanol undergoes a change in mechanism for the majority of the other determinations when it can be of a high negative value, which can be taken as evidence for a change in mechanism.

When solvent nucleophilicity is also an important factor, a scale of solvent nucleophilicity is developed, and a second term is added to the original Grunwald–Winstein equation, which is presented as Equation (2).

log (*k/k_o_*) = *lN* + *mY* + *c* (2)

The additional *lN* term incorporated effects controlled by the nucleophilicity of the solvent (ability to act as a nucleophile or as a base) leading to an *N* scale and the sensitivity of the rate to changes in its value governed by the *l* parameter. Ideally, a wide range of hydroxylic solvents is chosen, including solvents rich in a low nucleophilicity and high electrophilicity component, such as fluoroalcohol rich mixtures with water or ethanol. Such solvents will have high electrophilicity accompanying the low nucleophilicity. When combined with the original solvents, such as aqueous-ethanol, aqueous-methanol, and aqueous-acetone, the overall situation is good for avoiding multicollinearity when Equation (2) is used for a correlation analysis of the data [16,17]. The solvents almost always used are 2,2,2-trifluoroethanol (TFE) and, even more extreme in terms of low nucleophilicity and high ionizing power, 1,1,1,3,3,3-hexafluoro-2-propanol (HFIP). In addition to mixtures with water, mixtures of TFE with ethanol are frequently used (T-E). For historical reasons, the TFE-H_2_O and the HFIP-H_2_O mixtures are on a weight–weight basis and not the more used volume–volume (at 25 °C).

It was subsequently shown that the *tert*-butyl chloride choice as the substrate for establishing the original *Y* scale [15,17] was not ideal due to a moderate dependence of this solvolysis upon nucleophilic assistance from the solvent [16,18,19] (a low *l* value in the 0.1 to 0.2 region [16]). Additionally, it was found that, while the *Y*_Cl_ scale (developed with a chloride ion leaving group) was a good scale for general use, frequently better correlations for a leaving group X were obtained using *Y*_X_ scales, set up specifically for a leaving group X from solvolysis of an RX substrate [18,19].

**Scheme 3:**
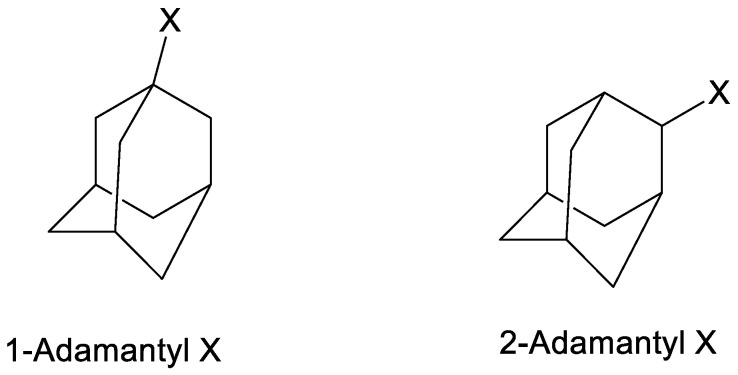
Adamantane derivatives typically used for the development of *Y*_X_ scales.

The structures of the cage adamantane derivatives are shown in Scheme 3 for the two types of monosubstitution that are possible. Here, it is obvious that the 1-adamantyl derivatives cannot react by an S_N_2 reaction because the back of the C-X bond is blocked by the cage, which would have to open and reform for such a substitution to occur. It is less obvious why an S_N_2 reaction cannot readily operate for the 2-adamantyl substrates, but a model of the inversion required for reaction shows that, at the transition state, hydrogens attached to the rings block the linear arrangement required for the favored S_N_2 transition state structure as regards the placing of the entering and the leaving groups [18,19]. Accordingly, the substitution reactions of a group attached to an adamantane carbon can be considered as proceeding by an “enforced” S_N_1 process. Further, E1 reaction (unimolecular elimination) would require a double bond at a bridgehead, which is also energetically disfavored (Bredt’s Rule).

Since substitutions (and/or eliminations) often occur with leaving groups other than chloride, several *Y*_X_ scales of leaving group ability have been developed. For poor leaving groups, the more reactive 1-adamantyl derivative is solvolyzed, and for the better leaving groups, the about 10^5^ times less reactive 2-adamantyl derivative can be used. The *p*-toluenesulfonates (tosylates) are frequently used since these can be prepared from the appropriate alcohol and *p*-toluenesulfonyl (tosyl) chloride [18,19]. Scales for a wide variety of X groups leaving from either 1-adamantyl or 2-adamantyl derivatives, whichever is more convenient, are available [19].

As regards scales of solvent nucleophilicity, the most successful of initial attempts to set up a scale was by applying Equation (2) to the solvolysis of methyl tosylate [18,19]. A value was needed for the *m* value in order to assess the relative importance of the leaving group in the presence of nucleophilic attack, where the scale of solvent nucleophilicity is obtained from Equation (3).

*N*_OTs_ = log (*k/k_o_*) – *mY*_OTs_ (3)

Using an estimated value of 0.30 for *m* [19], it was possible to obtain values for solvent nucleophilicity designated as *N*_OTs_.

**Scheme 4:**
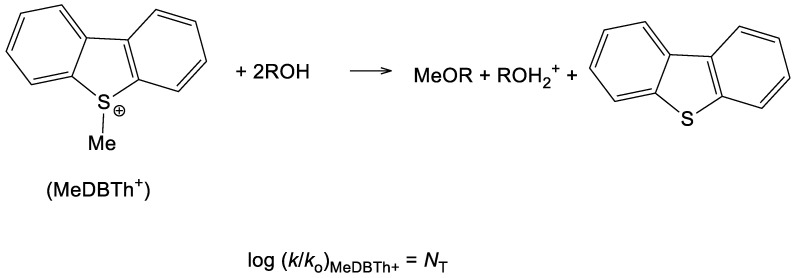
*N*_T_ scale is based on the solvolysis of *S*-methyldibenzothiophenium ion.

An alternative approach followed from the observation that, over the range of solvents usually employed in extended Grunwald–Winstein equation treatments, the specific rates of S_N_1 solvolyses of the 1-adamantyldimethylsulfonium ion were almost independent of the identity of the solvent [20]. This led to the rates of solvolysis of *S*-methyldibenzothiophenium ion being used directly (without the need for any adjustment) to set up a scale of solvent nucleophilicities designated as the *N*_T_ scale (Scheme 4).

In Scheme 4, *k* and *k*_o_ are the specific rates of solvolysis in a given solvent and in 80% ethanol, respectively, and *N*_T_ is the scale of solvent nucleophilicity values [21,22]. Additional values have been determined [23].

On the occasion of the 60^th^ anniversary of the publication [15] introducing the Grunwald–Winstein equation, a brief review of the history and the current applications of simple and extended forms was presented [24].

## 2. Results and Discussion

In Table 1 are listed the specific rates of solvolysis, together with the standard deviations, for 3-chloropropyl chloroformate (**4**), butyl chloroformate (**5**), and 4-chlorobutyl chloroformate (**6**), obtained as described in the “Materials and Methods” section of the manuscript. The solvents consisted of ethanol and methanol, mixtures of water with methanol, ethanol, acetone, 2,2,2-trifluoroethanol (TFE), and 1,1,1,3,3,3-hexafluoro-2-propanol (HFIP) plus TFE-ethanol mixtures (T-E).

Examining the specific rate values listed in Table 1, we can see that, for 100% ethanol through to 70% acetone, the presence of the terminal chlorine atom in **6** relative to the unsubstituted **5** leads to small increases in the specific rate value. This is consistent with the presence of the remote electron-withdrawing chlorine slightly reducing the electron density at the carbon bonded to the chlorine leaving group that leaves as a chloride ion, which favors an increased efficiency of the nucleophilic attack (Scheme 1).

In contrast, for the same chloroformate esters undergoing reaction in 97%, 70%, and 50% TFE, solvents of considerable higher ionizing power, we see somewhat lower values for the specific rates of solvolysis in the presence of the 4-chloro substituent. This can be rationalized in terms of the withdrawal of electrons from the reaction site leading to a higher reaction barrier and a slower reaction due to the electron-withdrawing influence of the chlorine substituent (Scheme 2).

In Table 2, correlations of the reaction rates for **4**, **5,** and **6** are presented in terms of the extended Grunwald–Winstein Equation (2) correlations, and *l* and *m* parameters are presented for overall correlations and for correlations of subsets believed to operate by the mechanisms presented in Scheme 1 and Scheme 2 when a change in mechanism from addition-elimination to ionization occurs as one goes across the spectrum for the influence of solvent properties on reaction mechanism. For comparison, previous correlations are presented to see how these new correlations contribute to the overall picture for the solvolysis of chloroformate esters.

Examination of Table 2 shows that, as already discussed earlier in this paper, the phenyl and the methyl esters avoid formation of the high energy methyl or phenyl cations and react by the addition-elimination mechanism over the full range of solvents. The ethyl chloroformate solvolysis also follows this mechanism for most of the studied solvolyses but, in the more ionizing fluoroalcohol-containing solvents, there is a second correlation with lower *l* value and higher *m* value, corresponding to a changeover to a dominant ionization mechanism. If a terminal chlorine atom is introduced into the ethyl group (compound **2**), a fairly good correlation is obtained when all the solvents are included, which shows appreciable improvement in the *R* value and slightly higher *l* value and slightly lower *m* value when the three points for the most ionizing solvents of the study (70% and 50% HFIP and 97% TFE) are omitted [1].

In addition to the solvolyses of 2-chloroethyl chloroformate, the solvolyses of the isomeric 1-chloroethyl chloroformate were also studied [25]. Here, the movement of the substituent closer to the reaction center increases its influence. If the data point for the most ionizing solvent of the study (90% HFIP) is removed, a good two term correlation is obtained, as reported in Table 2.

**Figure 1:**
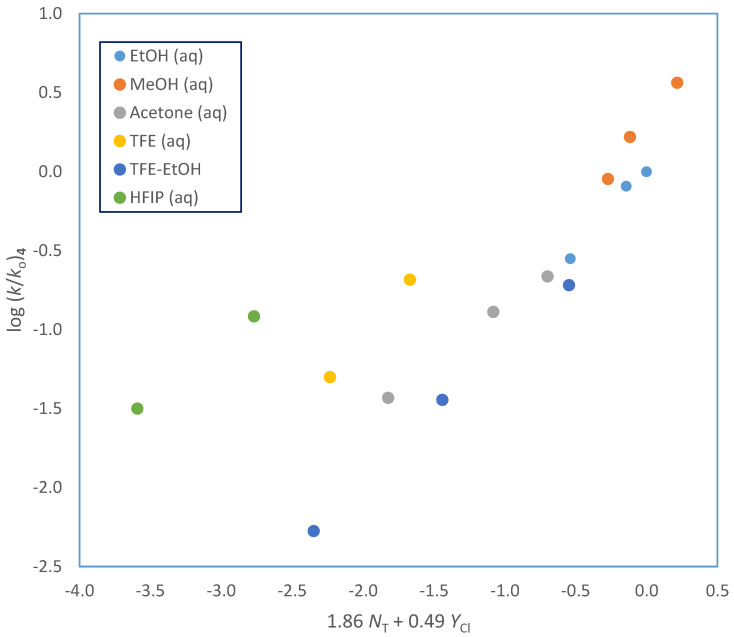
Plot of log (*k*/*k*_o_) for solvolyses for 3-chloropropyl chloroformate (**4**) against 1.86 *N*_T_ + 0.49 *Y*_Cl_ in twelve pure and binary solvents. The data points in TFE-H_2_O and HFIP-H_2_O are not included in the correlation; they are added to the plot to show the extent of their deviation.

The correlations for *n*-propyl chloroformate [3], as one would expect, parallel those for the ethyl chloroformate study [1], with the majority of the points corresponding to the operation of the addition-elimination pathway. In this study, the 3-chloropropyl chloroformate (**4**) with all measurements included gives a rather poor overall correlation (*R* value of 0.874, *F*-test value of 21), which improves considerably when the two data-points in TFE-H_2_O and in HFIP-H_2_O, the more highly ionizing solvents of the study, are excluded. The *R* value rises to 0.977, the *F*-test improves considerably to 93, and the *l* value rises from 1.39 to a more typical value for an addition-elimination pathway of 1.86. This behavior is demonstrated in Figure 1.

**Figure 2:**
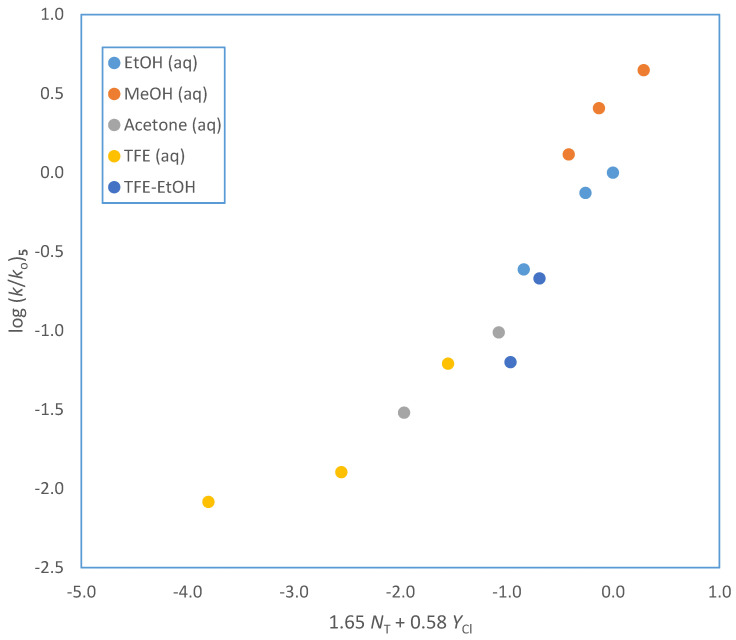
Plot of log (*k*/*k*_o_) for solvolyses for *n*-butyl chloroformate (**5**) against 1.65 *N*_T_ + 0.58 *Y*_Cl_ in eleven pure and binary solvents. The data points for 97% and 90% TFE are not included in the correlation; they are added to the plot to show the extent of their deviation.

**Figure 3:**
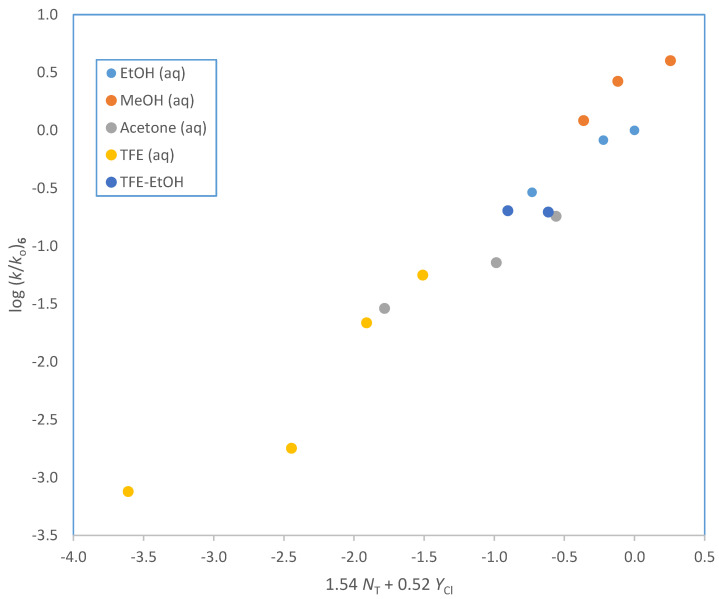
Plot of log (*k*/*k*_o_) for solvolyses for 4-chlorobutyl chloroformate (**6**) against 1.54 *N*_T_ + 0.52 *Y*_Cl_.

Similarly, in the present study, the specific rates reported for *n*-butyl chloroformate (**5**) give a correlation corresponding to the operation of the addition-elimination pathway if the data points for the strongly ionizing 97% and 90% TFE solvents are omitted. This behavior is demonstrated in Figure 2. On the other hand, for 4-chlorobutyl chloroformate (**6**), an inclusion of all the data points, as shown in Figure 3, results in an *F*-test = 100 with a good correlation *R* = 0.971, an *l* value of 1.54, and an *m* value of 0.52.

**Figure 4:**
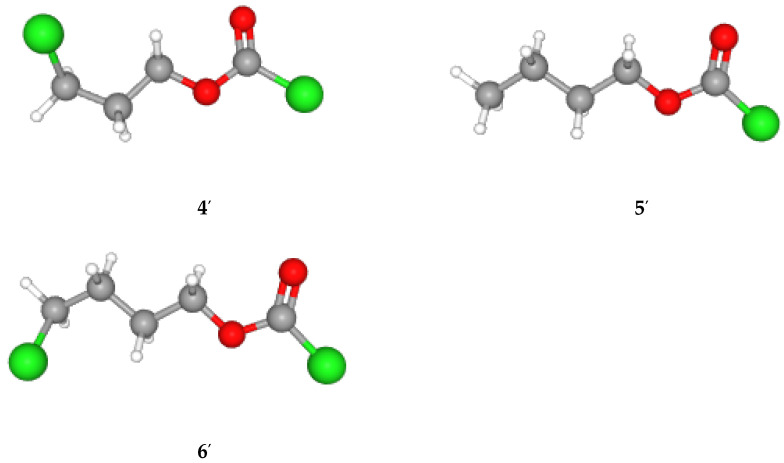
3D conformers of 3-chloropropyl chloroformate (**4^’^**), CID=69399, butyl chloroformate (**5^’^**), CID=61140, and 4-chlorobutyl chloroformate (**6^’^**), CID=2733328, from the National Center for Biotechnology Information. PubChem Database. 4-Chlorobutyl chloroformate, (accessed on 17 May 2020).

The PubChem Database’s 3D conformers for 3-chloropropyl chloroformate (**4^’^**), butyl chloroformate (**5^’^**), and 4-chlorobutyl chloroformate (**6^’^**) are shown in Figure 4. As a result of the proximity of the inductive chlorine substituent in **4** as compared to the reaction center in **6**, we observe *k***_4_** > *k***_6_** (Table 1) in all of the common aqueous ethanol, aqueous methanol aqueous acetone, aqueous TFE, and T-E mixtures. For **5** and **6**, the rate trend *k***_6_** > *k***_5_** is observed in common aqueous ethanol, aqueous methanol aqueous acetone, and T-E mixtures, as the electron-withdrawing chlorine atom in **6** facilitates the addition-elimination process. However, in the more ionizing aqueous 97% and 90% TFE solvents, *k***_5_** > *k***_6_**, which is consistent with the proposed more dominant ionization proposal for **5**.

The Table 2 solvents where the addition-elimination mechanism is indicated to be the dominant process have *l*/*m* ratios for **4**, **5,** and **6** of 3.80, 2.84, and 2.96, respectively. Such ratios lie within the *l*/*m* ratio range observed for other chloroformate esters [24] and demonstrate the importance of bond formation in the rate-determining tetrahedral transition state. The similarity of *l*/*m* ratios suggests that there is no direct participation by the terminal chlorine atom. However, the significantly higher ratio of 3.80 obtained for **4** in conjunction with the shape of its **4**^’^ (Figure 4) favored 3D conformer suggests a looser tetrahedral transition state with a greater catalytic presence of general base catalysis. For the secondary isopropyl chloroformate [26], one would expect, relative to primary alkyl chloroformate esters, an increased sensitivity to changes in solvent ionizing power coupled with a decreased sensitivity to changes in solvent nucleophilicity.

Consistent with that prediction, of a total of 25 solvolyses at 40 °C, it was found that nine favored an addition-elimination pathway (ethanol-water, methanol-water, and acetone water) with values for *l* of 1.35 ± 0.12 and for *m* of 0.40 ± 0.03, and sixteen favored an ionization pathway (fluoroalcohol-containing solvents), with values for *l* of 0.28 ± 0.04 and for *m* of 0.59 ± 0.04 and with *R* values of 0.960 for the addition-elimination pathway (Scheme 1) and of 0.982 for the ionization pathway (Scheme 2).

Consistent with the proposed change in the mechanism from addition-elimination to ionization as the solvent ionizing power value (*Y* value) increases, Crunden and Hudson [27] found, for chloroformate esters solvolyzing in 65% acetone, a rate order of Me > Et < *i*-Pr, consistent with the changes in mechanism outlined earlier. They also found, for solvolyses in formic acid (HCOOH), a highly ionizing solvent with a *Y*_Cl_ value of 3.20 essentially identical to that for 50% TFE [19], a change in the rate order to Me < Et < *i*-Pr, where both ethyl and isopropyl chloroformates must be reacting predominantly by the ionization mechanism

## 3. Materials and Methods

The 3-chloropropyl chloroformate (**4**, Sigma-Aldrich, St. Louis, MO, USA, 97%), the butyl chloroformate (**5**, Sigma-Aldrich, St. Louis, MO, USA, 98%), and the 4-chlorobutyl chloroformate (**6**, Sigma-Aldrich, St. Louis, MO, USA, 99%) were used as received. Solvents were purified as previously described [28]. The kinetic runs were carried out by removal of initial and final (at about ten half-lives) aliquots and titration of the developed acid after quenching in cooled acetone containing Lacmoid (Resorcinol Blue) as an indicator. The calculated specific rates (first-order rate coefficients) are presented, together with their associated standard deviations, in Table 1.

Multiple regression analyses of the specific rates, allowing for a comparison of the influence of changes in solvent nucleophilicity and solvent ionizing power in a range of solvents, were carried out. The solvents were chosen so as to give appreciable variations in the values for those properties, and the specific rates obtained were analyzed in terms of the extended Grunwald–Winstein equation (Equation (2)). The correlation details are presented, together with the results of earlier studies to allow for comparisons, in Table 2. The *l* and the *m* values included in the Table give a measure of the importance of solvent nucleophilicity and solvent ionizing power in the substitution process. The *N*_T_ and the *Y*_Cl_ values are from earlier tabulation [16,19,21,22,25,26,27]. The statistical analyses were carried out using the Excel 2016 package from the Microsoft Corporation.

## 4. Conclusions

In association with the proposal of a dominant addition-elimination mechanism occurring in the common and the more nucleophilic aqueous ethanol, aqueous methanol, aqueous acetone, and TFE-EtOH solvents, the observed (Table 1) rate trends for 3-chloropropyl chloroformate (**4**), butyl chloroformate (**5**), and 4-chlorobutyl chloroformate (**6**) are *k***_4_** > *k***_6_** > *k***_5_**. This direction changes to *k***_4_** > *k***_5_** > *k***_6_** in the less nucleophilic 70% aqueous TFE where an ionization process is suggested for **4**.

For any given solvent with available *N* and *Y* values, the calculated *l* and *m* sensitivity values (Table 2) can easily be applied to Equation (2) to calculate a solvent’s theoretical rate of reaction. A comparison of the solvents measured experimental rate to its targeted theoretical value can then provide mechanistic information that is critical and not otherwise obvious. For the very low nucleophilic and strongly hydrogen-bonding aqueous 97% TFE mixture, it was calculated (and this behavior is seen in Figure 2) that 98% butyl chloroformate (**5**) underwent ionization (Scheme 2), whereas only 33% of the 4-chlorobutyl chloroformate (**6**) followed a similar dissociation process (Figure 3). In aqueous 90% TFE, the ionization process for **5** was found to be 78%, and in 70% TFE, 88% of **4** underwent ionization.

## Figures and Tables

**Table 1:** Specific rates of solvolysis at 25 °C (***k***) for propyl chloroformate (**3**), 3-chloropropyl chloroformate (**4**), butyl chloroformate (**5**), and 4-chlorobutyl chloroformate (**6**) in various solvents and their literature values for *N_T_* [22] and *Y_Cl_* [19].

Solvent*^a^*	10^5^ *k*·s^−1^ (3)*^b,c^*	10^5^ *k*·s^−1^ (4)*^b^*	10^5^ *k*·s^−1^ (5)^b^	10^5^ *k*·s^−1^ (6)^b^	*N_T_^d^*	*Y_Cl_^e^*
100% EtOH	2.20 ± 0.03	6.36 ± 0.07	1.73 ± 0.09	3.56 ± 0.02	0.37	−2.50
90% EtOH	5.64 ± 0.04	18.3 ± 1.1	5.26 ± 0.14	10.1 ± 0.4	0.16	−0.94
80% EtOH	7.92 ± 0.05	22.6 ± 1.3	7.08 ± 0.17	12.2 ± 0.3	0.00	0.00
100% MeOH	8.88 ± 0.05	20.3 ± 1.5	9.21 ± 0.18	14.9 ± 1.3	0.17	−1.17
90% MeOH	18.8 ± 0.2	37.4 ± 1.3	18.1 ± 0.7	32.5 ± 1.4	−0.01	−0.18
80% MeOH	27.7 ± 0.3	82.5 ± 1.6	31.5 ± 1.4	48.9 ± 3.1	−0.06	0.67
90% Acetone	0.307 ± 0.003	0.837 ± 0.021	0.214 ± 0.010	0.354 ± 0.017	−0.35	−2.22
80% Acetone	0.942 ± 0.007	2.92 ± 0.14	0.689 ± 0.029	0.881 ± 0.012	−0.37	−0.83
70 % Acetone	1.91 ± 0.01	4.91 ± 0.14		2.21 ± 0.19	−0.42	0.17
97% TFE	0.0650 ± 0.004		0.0583 ± 0.009	0.0092 ± 0.0005	−3.30	2.83
90% TFE	0.0935 ± 0.0002		0.0899 ± 0.0015	0.0220 ± 0.0022	−2.55	2.85
70% TFE	0.591 ± 0.005	1.13 ± 0.04	0.438 ± 0.016	0.265 ± 0.011	−1.98	2.96
50% TFE	1.92 ± 0.02	4.68 ± 0.14		0.685 ± 0.009	−1.73	3.16
80T-20E	0.0666 ± 0.0003	0.120 ± 0.010			−1.76	1.89
60T-40E	0.342 ± 0.002	0.811 ± 0.052			−0.94	0.63
50T-50E			0.446 ± 0.010	0.721 ± 0.007	-0.64	0.16
20T-80E	1.60 ± 0.02	4.32 ± 0.15	1.51 ± 0.06	2.40 ± 0.09	0.08	−1.42
70% HFIP	0.499 ± 0.014	0.714 ± 0.011			-2.94	3.83
50% HFIP	1.15 ± 0.02	2.74 ± 0.13			-2.49	3.80

*^a^*Volume–volume at 25.0 °C for aqueous ethanol, aqueous methanol, aqueous acetone, and 2,2,2,-trifluoroethanol-ethanol (T-E) and weight–weight for 1,1,1,3,3,3,-hexafluoro-2-propanol (HFIP)-water mixtures and 2,2,2-trifluoroethanol (TFE)-water mixtures; *^b^*with associated standard deviations; *^c^* abstracted from [3]; *^d^* solvent nucleophilicity values (based on the solvolyses of the *S*-methyldibenzothiophenium ion) from [22]; *^e^*solvent ionizing power values (based on the solvolyses of 1-adamantyl chloride) from [19].

**Table 2:** Correlations at 25 °C using the extended Grunwald–Winstein equation (Equation (2)) of the specific rates of solvolysis of alkyl, monochloroalkyl, and phenyl chloroformates and classification in terms of the dominant mechanism addition-elimination (A-E) or ionization (I).

Substrate	*n*	*l*	*m*	*c*	*R*	*F*	Dominant Mechanism
PhOCOCl	49*^a^*	1.66 ± 0.05	0.56 ± 0.03	0.15 ± 0.07	0.980	568	A-E
MeOCOCl	19*^b^*	1.59 ± 0.09	0.58 ±0.05	0.16 ± 0.07	0.977	171	A-E
EtOCOCl (1)	28*^c^*	1.56 ± 0.09	0.56 ± 0.03	0.19 ± 0.24	0.967	178	A-E
EtOCOCl (1)	7*^c^*	0.69 ± 0.13	0.82 ± 0.16	−2.40 ± 0.07	0.946	17	I
Cl(CH_2_)_2_OCOCl (2)	19*^d^*	1.57 ± 0.21	0.61 ± 0.13	0.14 ± 0.17	0.903	35	A-E
Cl(CH_2_)_2_OCOCl (2)	16*^e^*	1.72 ± 0.18	0.54 ± 0.09	0.13 ± 0.12	0.943	53	A-E
CH_3_CHClCOCl	18*^f^*	1.43 ± 0.15	0.38 ± 0.10	0.17 ± 0.13	0.963	96	A-E
*n*-C_3_H_7_OCOCl (3)	22*^g^*	1.57 ± 0.12	0.56 ± 0.06	0.15 ± 0.18	0.947	83	A-E
*n*-C_3_H_7_OCOCl (3)	6*^g^*	0.40 ± 0.12	0.64 ± 0.13	−2.45 ± 0.47	0.942	11	I
Cl(CH_2_)_3_OCOCl (4)	15*^h^*	1.39 ± 0.25	0.52 ± 0.12	0.08 ± 0.16	0.874	21	A-E
Cl(CH_2_)_3_OCOCl (4)	12*^i^*	1.86 ± 0.14	0.49 ± 0.06	0.12 ± 0.18	0.977	93	A-E
*n*-BuOCOCl (5)	11*^j^*	1.65 ± 0.21	0.58 ± 0.09	0.22 ± 0.12	0.940	30	A-E
Cl(CH_2_)_4_OCOCl (6)	15*^k^*	1.54 ± 0.13	0.52 ± 0.08	0.16 ± 0.11	0.971	100	A-E
*i*-PrOCOCl	9*^l,m^*	1.35 ± 0.12	0.40 ± 0.05	0.18 ± 0.07	0.960	33	A-E
*i*-PrOCOCl	16*^l,n^*	0.28 ± 0.04	0.59 ± 0.04	−0.32 ± 0.06	0.982	176	I

*^a^* From [5] and [11]. *^b^* From [14]. *^c^* From [2]. *^d^* From [1] using all solvents. *^e^* From [1] with omission of values in 97% TFE and 70% and 50% HFIP. *^f^* From Table 3 of [25] with the value in 90% HFIP omitted. *^g^* From [25]. *^h^* Calculated from Table 1 of this paper with omission of data point for 70% HFIP. *^i^* Omitting the four data points for TFE-H_2_O and HFIP-H_2_O. *^j^*This work, omitting the data points for 97% and 90% TFE. *^k^* This work. *^l^* Runs carried out at 40 °C. *^m^* Using the data points in 100%, 90%, 80% MeOH, 100%, 90%, 80% EtOH, and 90%, 80%, and 70% acetone. *^n^*Using data points for solvolyses in the remaining solvents after those in footnote *m* are removed.
